# Pre- and Post-surgical Poor Seizure Control as Hallmark of Malignant Progression in Patients With Glioma?

**DOI:** 10.3389/fneur.2022.890857

**Published:** 2022-05-16

**Authors:** Giada Pauletto, Annacarmen Nilo, Christian Lettieri, Lorenzo Verriello, Barbara Tomasino, Gian Luigi Gigli, Miran Skrap, Tamara Ius

**Affiliations:** ^1^Neurology Unit, S. Maria della Misericordia University Hospital, Udine, Italy; ^2^Clinical Neurology Unit, S. Maria della Misericordia University Hospital, Udine, Italy; ^3^Scientific Institute, Istituto di Ricovero e Cura a Carattere Scientifico (IRCCS) E. Medea, Dipartimento/Unità Operativa Pasian di Prato, Udine, Italy; ^4^Neurosurgery Unit, S. Maria della Misericordia University Hospital, Udine, Italy

**Keywords:** brain-tumor epilepsy, low-grade glioma, malignant progression, electrocorticography, seizure outcome

## Abstract

**Background:**

Regarding brain tumor-related epilepsy (BTRE), there is an increasing number of evidence about a relationship between epileptogenesis and oncogenesis. A recent study suggests a role of post-surgery seizure outcome on the survival of patients with low-grade glioma (LGG), underlying the need for a targeted and aggressive epilepsy treatment.

**Objective:**

This study aims at investigating the possible correlation between pre- and post-surgical seizure control and tumor progression in patients who underwent surgery for LGG.

**Methods:**

We performed a retrospective analysis of patients affected by LGGs and BTRE, in a single high-volume neurosurgical center. Seizure control was assessed before surgery and at 3 years of follow-up. Patients with histological progression in high-grade glioma (HGG) have been evaluated. Clinical features, pre-surgical electroencephalograms (EEGs), and electrocorticography (ECoG) have been analyzed.

**Results:**

Among 154 subjects, we collected 32 patients who presented a tumor progression in HGG during the follow-up period. The majority had poor seizure control both pre- and post-surgery, never being in Engel class Ia throughout the whole history of their disease. Almost all patients with poor seizure control had pathological ECoG recording. Clinical features of seizures did not correlate with seizure outcome. On the univariate analysis, the age, the post-operative Engel class, and the extent of resection (EOR) were the prognostic factors significantly associated with oncological outcome; nevertheless, on multivariate analysis, Engel class significance was not confirmed, and the only predicting factor were age and EOR.

**Conclusions:**

Although not confirmed on multivariate analysis, post-surgical seizure control could be a relevant factor to consider during follow-up of BRTE, in particular, when gross total resection is not achieved. Pathological findings on the ECoG may suggest a “hidden” propensity to malignant progression, strictly related to the persistent neuronal hyper-excitability. Further studies with longer follow-up period are needed to confirm our observations.

## Introduction

Brain tumors (BTs) are considered rare tumors accounting for 1–2% of all tumors in adult people. Seizures represent one of the most frequent presenting signs of gliomas, so that epileptic seizures contribute to glioma diagnosis and impair its evolution (1).

Patients affected by supratentorial gliomas develop brain tumor-related epilepsy (BTRE) with an incidence varying from 60 to 100%, according to tumor type, grade, and location (1–3).

Seizure outcome has become more and more relevant in the clinical management of patients with glioma, and nowadays, it has been recognized not only as a negative factor for quality of life of these patients (3–5), but also as a significant prognostic factor for survival (6).

There is an increasing number of evidence about a close relationship between epileptogenesis and oncogenesis. Not only gliomas induce the onset of seizures, but also the epileptic activity influences tumor growth and progression (7). Anatomically, low-grade gliomas (LGGs) infiltrate the cortex and subcortical white matter and slowly disrupt functional networks. Glioma-related glutamatergic activity has been demonstrated to promote epileptic discharges in tumor-surrounding tissue and simultaneously stimulate tumoral cell proliferation, migration, and invasion of health brain parenchyma, inducing neuronal death *via* calcium excitotoxicity (8, 9).

Although there are several mechanisms to explain seizures development in the setting of BT (10, 11), predicting whether a patient will develop refractory epilepsy or experience a more malignant disease course remains a challenge in the clinical setting (12). A recent study suggests a role of post-surgery seizure outcome on the survival of patients with LGG, underlying the need for a targeted and aggressive epilepsy treatment (6).

In this study, we investigated the possible correlation between pre- and post-surgical seizure control and tumor progression in patients who underwent surgery for LGGs.

## Materials and Methods

### Study Population

We performed a retrospective analysis of 154 consecutive patients who presented a newly diagnosed supratentorial LGG with seizures as clinical presentation, in a single high-volume neurosurgical center (University Hospital of Udine, Italy). These patients underwent surgery between January 2007 and May 2018. Follow-up was extended until November 2021.

Patients were enrolled according to the following criteria:

- Age ≥ 18 years
- Pre-operative magnetic resonance imaging (MRI) suggestive of supratentorial LGG, confirmed by histology [according to the WHO 2016 classification (13)]
- One or more epileptic seizures as the clinical presentation of the glioma with a consequent diagnosis of BTRE
- No previous surgery
- No pre-operative chemo- or radiotherapy
- Objective evaluation of the extent of resection (EOR) on MRI in Digital Imaging and Communications in Medicine (DICOM) format based on T2-weighted MRI sequences
- Histological progression in high-grade glioma (HGG) within the observational period.

Needle biopsies were excluded from the study.

The local ethics committee (Comitato Etico Unico Regionale del Friuli Venezia Giulia) approved this investigation (protocol N.0036567/P/GEN/EGAS, ID study 2540). Considering that the study was retrospective, written consent to participate in the study was not applicable. Written informed consent was obtained for surgery from all patients.

### Clinical Data

Clinical information was retrieved from medical records.

We collected the following data: sex, age, time at first and second surgery, tumor localization and side, seizure type and frequency, type and number of anti-seizure medications (ASMs), pre-operative electroencephalogram (EEG), EOR, first and second histological molecular class, intraoperative electrocorticography (ECoG), the presence of intraoperative seizures (IOSs), and post-surgery seizure outcome.

Histological progression on the specimen from the subsequent surgeries was recorded and it was defined as increased glioma grade. Malignant progression-free survival (MPFS), defined as the time between initial surgery and demonstration of higher-grade tumor on subsequent biopsies, was calculated during the follow-up period for each patient. In those patients who died before the second surgery, MPFS was calculated as the time between initial surgery and demonstration of gadolinium enhancement on follow-up imaging.

The 2017 ILAE classification was applied to classify seizures (14). For statistical analysis, seizures were dichotomized, according to ictal semeiology, in motor (tonic, atonic, clonic, myoclonic, and hypermotor) and non-motor (sensory, autonomic, emotional, and cognitive) seizures.

Seizure frequency was assessed before surgery and after surgery for every 3 months for the first year and every 6 months thereafter for 2 years.

Post-operative seizure outcome was defined following the Engel Classification of Seizures (15) and dichotomized into 2 classes: Engel class Ia (completely seizure-free) vs. Engel class > Ia.

Engel class categories were assigned on the bases of self-completed seizure diaries. Engel class at 1-, 2-, and 3-years follow-up was used for the analysis.

### Pre-operative EEG Recordings

Patients underwent a pre-operative EEG recording (32-channel EB Neuro Mizar Sirius system with Galileo NT software, EB Neuro) according to the 10–20 International System, within 7 days before surgery.

**Figure 1:**
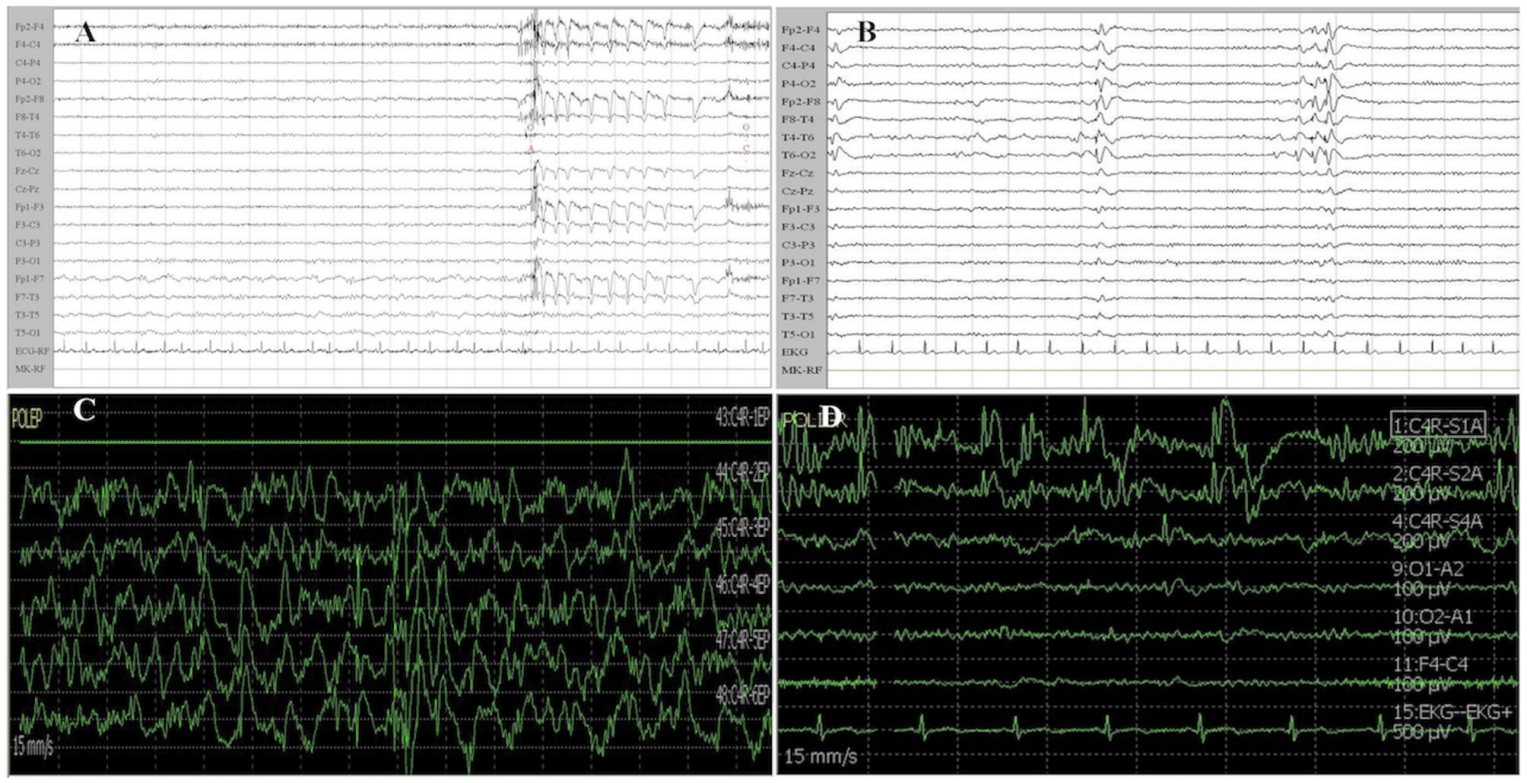
Examples of EEG and ECoG recordings from patients of the study cohort. **(A)** Patient 1 was affected by a left insular LGG. EEG recording shows a slow activity in delta band (1–2 Hz) mixed with an alpha background rhythm on the left frontotemporal regions. **(B)** Patient 2 suffered from a right temporal LGG. EEG shows interictal epileptiform activity characterized by spike-and-wave complexes on right temporal region (T4–T6 electrodes) which rapidly spread to the homolateral supra-sylvian region. **(C)** Patient 3 was affected by a right frontal LGG. ECoG traces recorded from a contact subdural strip located near the Rolandic region show a high amplitude diffuse and continuous slow activity (delta band). **(D)** Patient 1 was affected by left insular glioma (the same patient of **A**). ECoG traces (1, 2) recorded near the insular region show epileptic activity characterized by high amplitude spike-and-wave complexes. Other ECoG traces present low amplitude theta–alpha activity. ECoG gain 400 μV/div, time base 15 mm/s, bandpass 1–80 Hz. EEG gain 100 μV/cm, time base 15 mm/s, and bandpass 1–70 Hz. ECoG, electrocorticography; EEG, electroencephalography.

EEGs were scored as follows:

- Normal (N): background activity with alpha or faster rhythms, no focal or diffuse slowing, no epileptic discharges;
- Slow (S): alpha or faster rhythms as background with focal or multifocal slow activity, or alpha rhythm mingled with diffuse theta–delta activity (Figure 1A). Epileptic activity was absent;
- Epileptic (E): alpha activity in the background with faster rhythms or mixed with slower activity. Localized or diffused interictal epileptiform abnormalities (spikes, polyspikes, spike-and-wave, polyspike-and-wave complexes) were present (Figure 1B).

### Surgical Procedure

All patients underwent awake surgery following the standard protocol previously described (16). When necessary, general anesthesia was performed. The surgical procedures were conducted under cortical and subcortical white matter brain mapping, according to the previously reported intraoperative technique (17).

### Anesthetic Protocol

Total intravenous anesthesia with Propofol and Remifentanil infusions was used for patients operated under general anesthesia.

In the case of awake surgery, Remifentanil was used at a median dose of 0.02 μg/kg/min. The scalp was injected with local anesthetic (20 ml 2% lidocaine). Low doses of Propofol were allowed only at the end of surgery. Mannitol 18% 0.25–0.5 g/kg was administered in the case that the neurosurgeon complained of severely impaired brain relaxation.

### Intraoperative Electrocorticography

Electrocorticography was recorded using a 32-channels device (Axon System Eclipse^®^) and carried out by the experienced neurophysiologists. Recordings were analyzed separately offline by two neurophysiologists (G.P. and C.L.). In the case of discordance, a final review of ECoG traces was performed by a third neurophysiologist (A. N.). Recordings started before resection by placing 2–3 subdural strip electrodes over and around the lesion. During surgery, the strips were placed on the margin of the exposed area. The reference electrode was located on the forehead (Fpz).

The low-frequency filter (LFF) was set at 1 Hz, the high-frequency filter (HFF) at 80 Hz, and sensitivity was set between 300 and 500 μV/mm, according to the amplitude of background and epileptiform activity. A simultaneous EEG was acquired, with the following reduced montage: O1-Pz, O2-Pz plus F3-C3 or F4-C4 plus P4-O2 or P3-O1 depending on the tumor side. LFF was set at 1 Hz, and HFF was set at 70 Hz.

ECoG recordings were scored as follows:

- Normal (N): background activity with alpha or faster rhythms, with no epileptic discharges and slow activity;
- Slow (S): background alpha or beta rhythms with focal or multifocal slow activity, but no epileptic discharges (Figure 1C);
- Epileptic (E): alpha or slow activity in the background with focal or diffuse interictal epileptiform activity (Figure 1D), which is described according to the classification of Palmini et al. (18).

Intraoperative seizures were defined as any seizure observed during surgery. If no detectable clinical sign was witnessed, the seizure was described as electrographic; otherwise, the seizure was scored as electro-clinical. Spontaneous ECoG/EEG ictal activity was defined as evolving discharges characterized by one of the following patterns: rhythmic waves (in theta, delta, or alpha bands), rhythmic spiking, repetitive spike/polyspikes-waves or electro-decremental pattern, represented by a general attenuation of background rhythms which are substituted by low-voltage, high-frequency activity (19). These patterns were characterized by an abrupt onset, a clear evolution in amplitude, frequency, and/or topography over time and must last at least 10 s (20). Similarly, stimulation-induced seizures were defined as trains of after-discharges that evolved in terms of distribution, morphology, and/or frequency (21).

### Statistical Analysis

Descriptive analysis of the main features of the study population was performed using mean ± SD or median and range for continuous variables, and percentages for categorical variables. For the statistical analysis, we considered the oncological progression (i.e., the malignant transformation) as the function of the MPFS. The *t*-test or Mann–Whitney U-test, as appropriate, was used to compare continuous variables between groups. For categorical variables, cross-tabulations were generated, and a chi-square or Fisher's exact test was used to compare distributions, as appropriate. Survival was analyzed by means of Cox regression method.

In univariate analysis, the variables considered as possible prognostic factors were as follows: age, sex, post-operative Engel class, pre-operative EEG (epileptiform vs. not epileptiform), pre-operative seizures frequency, pre-operative seizure semiology and duration, ASMs, intraoperative ECoG data (epileptiform vs. not epileptiform), and the presence of IOS and EOR.

To assess the potential impact of missing data on the long-term results, the last observation carried forward (LOCF) analysis was performed. The seizure frequency at the last observation was carried forward for dropouts and used to impute the missing values. The combination of the observed and imputed data was then analyzed as though there were no missing data. After 3 years of follow-up, Engel class data were too numerically limited to perform a reliable LOCF analysis, so they were not considered in the study.

The results are presented as hazard ratios and 95% confidence intervals. All analyses were conducted using STATA/SE (version 14.0 Stata Corp.) for Windows. All two-tailed statistical significance levels were set at *p* < 0.05. Covariates with *p* < 0.05 at univariate analysis were selected for multivariate stepwise analysis.

## Results

A total of 154 patients affected by LGGs with seizures as clinical manifestations have been evaluated. In Table 1, demographic, clinical, and neurophysiological data are reported.

**Table 1:** Baseline characteristics of the study population.

**Variables**	
**No. of patients**	154
**Sex**, ***n*** **(%)**	
Male	95 (61.68)
Female	59 (38.32)
**Age, (years)**	
Median (IQR)	37.00 (58)
Range	15–73
**Seizure onset**	
Focal seizures	66 (42.86)
Focal to bilateral tonic–clonic seizures	88 (57.14)
**Seizure types**
Motor	105 (68.18)
Non-motor	49 (31.82)
autonomic	9 (5.80)
cognitive	13 (8.40)
sensory	18 (11.70)
emotional	9 (5.80)
**Pre-operative seizures frequency**	
Monthly	92 (59.74)
Weekly	51 (33.12)
Daily	11 (7.14)
**ASMs regimen**	
Monotherapy	126 (81.82)
Levetiracetam	91 (72.22)
Sodium channel blockers	24 (19.05)
Valproic acid	7 (5.50)
Phenobarbital	3 (2.38)
Zonisamide	1 (0.85)
Polytherapy	28 (18.18)
**Pre-operative EEG features**	
Normal	71 (46.10)
Slow	43 (27.92)
Epileptic	40 (25.98)
**Tumor side**	
Left	89 (57.10)
Right	66 (42.90)
**Tumor site**	
Frontal	52 (33.80)
Parietal	14 (9.10)
Temporal	24 (15.60)
Insular	64 (41.60)
**Pre-operative tumor volume (T2-weighted MRI**	
**images – cm** ^**3**^ **)**	
Median	48
Range	(6–144)
**EOR % (range)**	88 (38–100)
**Molecular Class**	
Oligodendroglioma IDH1/2 mutated 1p-19q codeleted	44 (28.60)
Diffuse astrocytoma IDH1/2 mutated 1p-19q non codeleted	92 (59.70)
Diffuse astrocytoma IDH1/2 wild-type	18 (11.70)
**MGMT promoter methylation**	
Yes	135 (87.70)
No	19 (12.30)
**Time between seizure onset and first surgery (months)**	6 (4–20)
**Intraoperative seizures**	
Yes	38 (24.68)
No	116 (75.32)
**Intraoperative ECoG features**	
Normal	48 (31.15)
Slow	24 (15.65)
Epileptic	82 (53.20)
**Post-operative Engel class at 1 year**	
Ia	108 (70.13)
>Ia	46 (29.87)
**Post-operative Engel class at 2 years**	
Ia	104 (67.53)
>Ia	50 (32.47)
**Post-operative Engel class at 3 years**	
Ia	99 (64.28)
>Ia	55 (35.72)

*ASMs, anti-seizure medications; ECoG, electrocorticography; EEG, electroencephalogram; EOR, extent of resection; IDH, isocitrate dehydrogenase; IQR, interquartile range; MGMT, O(6)-methylguanine-DNA methyltransferase*.

*Patients' characteristics are described using median and range for continuous variables, the number of cases with relative percentages (in parentheses) for categorical variables*.

Regarding epilepsy characteristics, the majority of patients experienced focal to bilateral tonic–clonic seizures (57.14%), while the remaining 66 patients (42.86%) suffered from focal seizures. Pre-surgery, seizures recurred daily in 11 patients (7.14%), weekly in 51 (33.12%), and monthly in 92 patients (59.74%). The most used ASM regimen was monotherapy (126 patients, 81.82%).

Pre-operative EEG showed no abnormalities or only slow activity (focal or bilateral) in the majority of patients (114, 74.02%). Intraoperatively, epileptic and not epileptic abnormalities were almost equally represented as shown by ECoG (72 patients vs. 82 patients, respectively). The majority of patients did not show any IOS (116, 75.32%).

Then, 1 year post-surgery, all patients completed seizure diaries: the majority of them (108, 70.13%) were in Engel class Ia. At 2 and 3 years post-surgery follow-up, the cohort that completed diaries included 110 and 87 patients, respectively. Missing data were due to the loss of follow-up and/or patients' death.

During the 3 years of follow-up (from 2018 to 2021), 32 patients presented a histological or radiological progression into HGG. Median MPFS was 70.5 months with a range of 6–239 months. The majority of them (67.8%) had poor seizure control both pre- and post-surgery, never being in Engel class Ia throughout the whole history of their disease. Considering pre-surgery seizure frequency, they presented daily or weekly attacks. All patients with poor seizure control had pathological ECoG recording, particularly about 60% showed an epileptic ECoG.

Seizure characteristics did not differ significantly between patients with HGG who were seizure-free and patients with HGG who were not.

The univariate analysis by means of Cox regression (Table 2) showed that the covariates associated with oncological outcome were as follows: age, post-operative Engel class, and EOR. Indeed, at 1-year post-surgery, we observed that the majority of patients with no evidence of histological progression (90, 73.78%) were in Engel class Ia with a statistically significant correlation (*p* < 0.01). Then, 2 and 3 years post-surgery, we observed a stronger association between Engel class Ia and the absence of progression with high levels of statistical significance (*p* < 0.001), regardless of the type of analysis performed (observed data plus LOCF vs. observed data only). Nevertheless, on multivariate analysis, the only independent predictor factors associated with the oncological outcome were age and EOR, as observed by the previous studies (22), whereas Engel class significance was not confirmed.

**Table 2:** Predictors of the oncological outcome on univariate and multivariate analysis by means of Cox regression.

		**MPFS**			**MPFS**		
		**Univariate analysis**			**Multivariate analysis**		
		**HR**	**95% CI**	***p***	**HR**	**95% CI**	***p***
**Clinical feature**	**Reference variable**						
Sex	Male	0.9492	0.6292–1.4319	0.8038			
Age^§^		1.0303	1.0121–1.0489	**<0.01**	1.0238	1.0050–1.0430	**0.0129**
**Pre-operative epilepsy features**							
Seizure type	Motor	0.9072	0.5919–1.3903	0.6548			
Seizure onset	Focal (incl. FTBTC)	0.7101	0.4762–1.0593	0.0934			
Seizure frequency	Monthly	1.1487	0.7689–1.7159	0.4985			
Duration	<1 year	1.0590	0.6102–1.8380	0.8385			
Pre-operative EEG	Not epileptiform	1.1122	0.7089–1.7449	0.6436			
ASMs	Monotherapy	1.6210	0.9887–2.6577	0.0555			
**Intraoperative features**							
ECoG	Not epileptiform	1.3127	0.8789–1.9607	0.1837			
Intraoperative seizures	None	1.0463	0.6670–1.6413	0.8438			
**Postoperative features**							
Engel class (1 year post-surgery)	Engel I	2.2633	1.4921–3.4332	**<0.01**	1.0911	0.5548–2.1457	0.8005
Engel class (2 years post-surgery)^*^	Engel I	2.2144	1.4737–3.3274	**<0.001**	1.0936	0.3424–3.4931	0.8800
Engel class (3 years post-surgery)^*^	Engel I	2.1617	1.4493–3.2421	**<0.001**	1.6769	0.5968–4.7172	0.3267
EOR (%)^§^		0.9598	0.9458–0.9741	**<0.0001**	0.9680	0.9519–0.9845	**<0.001**

§ *Modeled as continuous variable*.

* *Observed data plus LOCF (Last Observation Carried Forward)*.

*EEG, electroencephalogram; ASMs, anti-seizure medications; ECoG, electrocorticogram; MPFS, malignant progression-free survival*.

*Significant p-values are reported in bold*.

Demographic features, as well as pre-operative seizures characteristics and intraoperative data, were not statistically associated with oncological outcomes.

## Discussion

In this study, we investigated the potential role of post-surgical seizure outcome on tumor progression in a cohort of patients affected by LGGs and BTRE. We observed that poor post-surgery seizure control was potentially associated with tumor progression into HGG within 3-year follow-up, although not confirmed on multivariate analysis.

The extent of surgical resection is an established prognostic factor for seizure and oncological outcomes (22, 23). Thus, post-surgical persistence of seizures is often the consequence of an uncomplete resection of epileptogenic zone (EZ), even in the case of glioma surgery.

In fact, two scenarios may be observed: the EZ may lie away from the tumoral area or may be nestled within the residual tumor. In this context, an extended pre-surgical neurophysiological evaluation may be useful to better define the EZ and so to guide intraoperative monitoring, to maximize the EOR.

In our experience, the presence of interictal ECoG activity on surgical margins suggests a post-surgical seizure recurrence.

Moreover, the persistence of seizures after surgery could facilitate tumor progression not only because it is an indirect clue of an uncomplete resection, but also for the possible enhancement of oncogenetic process driven by seizures themselves.

In fact, the importance of seizure control in patients with gliomas is increasingly emerging. Our results are in line with this evidence.

Santos-Pinheiro et al. showed that a high post-surgical seizure frequency and an increase in seizure frequency from pre- to post-operative period were associated with a greater rate of early tumor recurrence in a LGG population (12). Furthermore, in another recent Italian work, seizure outcome after surgery emerged as an independent strong predictive factor of overall survival in patients with glioma (6).

In our study, we focused mainly on clinical and epileptological features for two reasons. First of all, neurosurgical and molecular characteristics associated with tumor progression or recurrence have already been extensively evaluated (23–28). In the last decades, this growing body of literature remarks as an extensive early surgery leads to obtain a good oncological and epileptological outcomes (23–26). Second, recent studies have pointed out that epileptogenesis and tumor growth in LGGs may share common pathogenetic mechanisms that can influence each other (28, 29).

In this context, an early, careful, and constant evaluation and management of seizures, both pre- and post-surgery, in patients with glioma, finds its rational.

In fact, after glioma resection, Neal et al. found a prevalence of fluctuating seizure control pattern in patients affected by grade II and III gliomas and BTRE (30). They interpreted this result as the consequence of the natural history of delayed but expected progression. Therefore, the first period of seizure freedom might be the result of removing the epileptogenic zone with a gross total resection, whereas seizure relapse might reflect tumor progression (3, 30, 31). Moreover, Mittal et al. performed intracranial EEG analyses on patients affected by glioma-related drug-resistant epilepsy and showed that seizure onset zone included tissue located beyond 1.5 cm from the tumor margin (32).

Taken together, all this evidence suggests that glioma surgery, at least in patients already affected by BTRE, should include, when possible, the resection of epileptogenic zone, removing peritumoral tissue where epileptic foci are more likely to be nested. In fact, seizures arise electrographically from the peritumoral cortex in most of the patients, due to induced changes rather than from the tumor proper (33).

The mechanisms of epileptogenesis in gliomas are multifactorial and some are also involved in neuronal death, changes in cellular mobility, and oncogene expression *via* second messengers. Among the epileptogenic pathways, it is of main importance the so-called glutamatergic one.

In peritumoral cortex, an increase in glutamatergic activity has been demonstrated (33, 34). In their experimental work, Buckingham et al. implanted human-derived glioma cells into combined immunodeficient mice. These glioma-bearing mice developed spontaneous and recurring epileptic activity, as a consequence of marked glutamate release from the tumor, mediated by the system x**c–** cystine–glutamate transporter (34).

Moreover, the high glutamate levels in tumor tissue are also a consequence of both increased release of a glutamate agonist in the synaptic cleft, induced by mutation of IDH 1/2 (isocitrate dehydrogenase 1 and 2) (9), and a reduced glutamate removal from extracellular space, caused by the downregulation of excitatory aminoacidic transporter EAAT2 (35).

Peritumoral astrocytes that would normally be able to remove and catabolize extracellular glutamate are overwhelmed by glutamate release from the tumor, and peritumoral neurons exhibit a lower epileptic threshold. Furthermore, glutamate release from glioma leads to tumor growth, tumor-associated excitotoxicity, tumor invasion of health parenchyma and edema (34).

Finally, Feyissa et al. performed a transcriptome-wide comparison between patients with glioma-related seizures (GRS), subjects with glioma but no seizures (non-GRS), and patients with idiopathic temporal lobe epilepsy (iTLE) (36). They found differential expressed genes associated with patients with GRS vs. non-GRS. Particularly, in the former group, there were a significant overexpression of genes involved in cell-to-cell and glutamatergic signaling (CELF4, SLC17A7, and CAMK2A) and a down-regulation of genes involved immune-trafficking (CXCL8, H19, and VEGFA). Comparing GRS with patients with iTLE, an overexpression of genes considered markers of oncogenesis was observed in the first group (36).

Thus, the post-surgical persistence of seizures may depend on the impossibility of removing the epileptogenic zone, the multiple pathogenetic mechanisms that are involved in seizure generations, and the activation of different epileptic networks. Epileptic firing might enhance oncogenesis by the amplification of common pathogenetic pathways.

We acknowledge that our study is retrospective and it carries all the intrinsic limitations of this study design. Furthermore, histological reports were classified according to the previous 2016 WHO classification of brain tumors (13). Thus, the prognostic role of CDKN2A/2B, ATRX, TERT, EGFR, and TP53 mutations emerged by the 2021 WHO Classification (37) was not assessed, explaining an overestimation of the real number of LGGs included in our study population. However, this study analyzes a homogeneous population (all patients with a first diagnosis of LGG and affected by BTRE from the beginning), with a long follow-up and it focuses on epileptological and electroencephalographic features, since patients have been evaluated by a multidisciplinary team including neurologists expert in epilepsy and clinical neurophysiology.

## Conclusions

Seizure control has major implications for the quality of life in patients with BRTE, as intractable seizures are associated with significant morbidity. In LGG population, the possibility that a poor seizure outcome may correlate with a histological progression corroborates the importance of an early, constant, and careful evaluation and management of seizures, considering also target therapy for BTRE, such as ASMs that could impair common pathogenic pathways. A closer follow-up for patients who are not seizure-free after surgery should include also prolonged EEG recordings, to evaluate subtle seizures.

## Data Availability Statement

The raw data supporting the conclusions of this article will be made available by the authors, without undue reservation.

## Ethics Statement

The studies involving human participants were reviewed and approved by Comitato Etico Unico Regionale del Friuli Venezia Giulia. Written informed consent for participation was not required for this study in accordance with the national legislation and the institutional requirements.

## Author Contributions

GP and AN: conception and design. AN, GP, CL, and LV: acquisition of data. CL: formal analysis. TI, GP, GLG, and MS: supervision. GP and TI: validation. GP, AN, and CL: writing–original draft. GP, TI, AN, BT, and LV: writing, reviewing and editing. All authors have read and agreed to the published version of the manuscript.

## Conflict of Interest

The authors declare that the research was conducted in the absence of any commercial or financial relationships that could be construed as a potential conflict of interest.

## Publisher's Note

All claims expressed in this article are solely those of the authors and do not necessarily represent those of their affiliated organizations, or those of the publisher, the editors and the reviewers. Any product that may be evaluated in this article, or claim that may be made by its manufacturer, is not guaranteed or endorsed by the publisher.

## Abbreviations

ASMs: anti-seizure medications
BT: brain tumor
BTRE: brain tumor-related epilepsy
DICOM: Digital Imaging and Communications in Medicine
EAAT2: excitatory aminoacidic transporter 2
ECoG: electrocorticography
EEG: electroencephalogram
EOR: extent of resection
EZ: epileptogenic zone
GRS: glioma-related seizures
HFF: high-frequency filter
HGG: high-grade glioma
IDH1/2: isocitrate dehydrogenase 1 and 2
ILAE: International League Against Epilepsy
IOS: intraoperative seizures
iTLE: idiopathic temporal lobe epilepsy
LFF: low-frequency filter
LGG: low-grade glioma
LOCF: last observation carried forward
MGMT: O(6)-methylguanine-DNA methyltransferase
MPFS: malignant progression-free survival
MRI: magnetic resonance imaging
SD: standard deviation
WHO: World Health Organization.
